# Arthroscopic capsular release for frozen shoulder: when etiology matters

**DOI:** 10.1007/s00167-023-07561-2

**Published:** 2023-09-13

**Authors:** Olimpio Galasso, Michele Mercurio, Francesco Luciano, Claudia Mancuso, Giorgio Gasparini, Massimo De Benedetto, Nicola Orlando, Roberto Castricini

**Affiliations:** 1 Department of Orthopaedic and Trauma Surgery, “Magna Græcia” University, “Mater Domini” University Hospital, V.le Europa, 88100 Catanzaro, Italy; 2 Division of Orthopaedic and Trauma Surgery, “Villa Verde”, 63900 Fermo, Italy

**Keywords:** Frozen shoulder, Adhesive capsulitis, Arthroscopy, Capsular release, Etiology, Pain, Range of motion, Constant-Murley score, Anxiety, Depression

## Abstract

**Purpose:**

No therapeutic intervention is universally accepted for frozen shoulder, and the most effective management to restore motion and diminish pain has yet to be defined. The aim of this study was to investigate functional and psychological outcomes in patients who underwent arthroscopic capsular release for a frozen shoulder.

**Methods:**

A retrospective study with prospective data collection was conducted with 78 patients suffering from frozen shoulder resistance to conservative treatment. Considering the etiology, there were 36 (46.2%) idiopathic, 31 (39.7%) postoperative, and 11 (14.1%) posttraumatic cases. Preoperatively, each patient was evaluated with the range of motion (ROM) assessment and the Constant-Murley score (CMS). At follow-up, the 4-point subjective satisfaction scale (SSS), the ROM assessment, the SF-12 questionnaire, the numerical rating scale (NRS) for the subjective assessment of pain, the CMS and the Hospital Anxiety and Depression Scale (HADS) were assessed.

**Results:**

After a mean follow-up of 54.2 ± 22.3 months, ROM and CMS showed a statistically significant improvement between pre- and postoperative values (all *p* < 0.001). Before surgery, the mean CMS was 36.9% that of sex- and age-matched healthy individuals, and all patients showed a CMS lower than the normative data. At the final follow-up visit, the mean CMS was 99.9% that of sex- and age-matched healthy individuals, and 49 (62.8%) patients showed a CMS equal to or higher than the normative data. The mean increase in the CMS was 56.1 ± 8.3 points. The mean SSS, HADS-A, HADS-D, and NRS were 3.7 ± 0.5, 2.5 ± 1.6, 2.2 ± 1.3, and 2.2 ± 1.0, respectively. All patients returned to their previous level of work and sports activity after 2 and 2.5 months, respectively. The multivariate analysis showed the association between a higher postoperative CMS and the idiopathic etiology of a frozen shoulder (*p* = 0.004, *β* = 3.971). No intraoperative complications occurred. Postoperatively, four patients (5.1%) were treated with intra-articular steroid injections to manage residual symptoms. One patient (1.3%) with a postoperative frozen shoulder showed persistent symptoms and underwent a new successful arthroscopic capsular release.

**Conclusion:**

High patient satisfaction and statistically significant ROM and CMS recovery can be achieved after arthroscopic capsular release to manage frozen shoulder. Better functional outcomes are expected when the etiology is idiopathic. Results can help surgeons identify the patients who will most benefit from surgery and should be discussed with the patient.

**Level of evidence:**

III.

## Introduction

Frozen shoulder was first defined by Codman, and the term is usually used to describe a patient who presents a restricted range of motion of the shoulder and chronic pain [16] that may limit an individual’s ability to work and perform activities of daily living [18]. The prevalence of frozen shoulder ranges from 2 to 5%, with a peak incidence between 40 and 60 years of age [15]. Shoulder stiffness can be either primary (idiopathic) or secondary to postoperative or posttraumatic conditions such as rotator cuff repair, proximal humerus fracture or neurological or thyroid pathologies [31]. While the pathophysiology of primary stiffness is not entirely clear, postoperative and posttraumatic frozen shoulder may be related to cuff tear morphology, immobilization, glenohumeral adhesion, capsular contracture, or underlying predisposing patient comorbidities [26]. Patients with frozen shoulder may struggle with daily activities and suffer anxiety, depression and sleep disturbance [28].

Although a frozen shoulder is often considered to be self-limiting, full resolution of symptoms does not always occur [28]. Currently, no therapeutic intervention is universally accepted, and the most effective management to restore motion and diminish pain has yet to be defined [21]. Most patients are initially prescribed physical therapy and corticosteroid injections prior to referral for surgery [3]. Although most cases of frozen shoulder respond to nonoperative management, surgery may become necessary in cases where conservative treatment has failed [12, 20]. The results of surgical management show favorable outcomes [18], and arthroscopic capsular release is an effective treatment across all etiologic groups of the frozen shoulder [25].

The aim of this study was to investigate functional and psychological outcomes in patients who underwent arthroscopic capsular release for the frozen shoulder. Variables that can predict outcomes were also investigated. The hypothesis is that patients with idiopathic stiffness have better outcomes after arthroscopic capsular release than those with frozen shoulder with a postoperative or posttraumatic etiology.

The study results could help surgeons identify the patients who will benefit most from surgery.

## Materials and methods

A retrospective study with prospective data collection was conducted with 85 patients suffering from frozen shoulder resistance to conservative treatment who underwent arthroscopic capsular release between 2014 and 2020. According to a recently published FROST trial, frozen shoulder was defined as a passive external rotation range of motion less than 50% of that of the opposite shoulder [28]. The study protocol was approved by the local ethics committee (Villa Verde Hospital, ID: 022021), and the research was conducted in compliance with the Declaration of Helsinki. The inclusion criteria were (1) idiopathic, postoperative or posttraumatic frozen shoulder with an intact rotator cuff, (2) persistent symptoms with pain and stiffness despite conservative treatment conducted for at least 4 months from the onset of stiffness, and (3) a minimum of 12 months of follow-up. Therefore, patients with previous fractures of the proximal humerus, clavicula, or scapula and patients who had already undergone shoulder surgery for rotator cuff tears, fractures, or instability were included in this study. The exclusion criteria were (1) infectious arthritis, (2) moderate-to-severe glenohumeral osteoarthritis, (3) neurological disorders of the upper extremities, (4) significant cognitive impairment, (5) a diagnosis of or treatment for any psychiatric condition, and (6) failure to understand or complete the questionnaires. The diagnosis was based on clinical (history, signs and symptoms) and radiological (X-rays and magnetic resonance imaging) evaluation of the shoulder. A magnetic resonance imaging scan was carried out to evaluate rotator cuff tears and any additional intra- and/or extra-articular glenohumeral joint abnormality suitable for different treatments. Informed consent was obtained from all participants included in the study. Preoperatively, all patients had shoulder pain, weakness, and functional limitations that interfered with their activities of daily living.

The collected data included the age of the patient, sex, dominant arm, etiology of the frozen shoulder, comorbidities, pre- and postoperative physical therapy, any other pre- or postoperative treatment, time to return to work and sports activities, and willingness to undergo the operation again.

### Surgical technique

The capsular release procedure was performed by the same surgeon (R.C.). The patient was placed in the lateral decubitus position, and in the absence of contraindications, locoregional anesthesia with an interscalene block was performed for all procedures. Care was taken to avoid articular cartilage injury during the insertion of the arthroscope through the tight capsule, initially via standard posterolateral access. A systematic inspection was performed to identify areas of synovitis and contracted tissue and any additional pathology. The surgical treatment started with the release of the rotator interval to the undersurface of the conjoint tendon, and the release was extended inferiorly posterior to the subscapularis tendon. Care was taken not to violate the subscapularis tendon. The superior release was then extended to reach the long head of the biceps and continued to release the coracohumeral ligament in the plane between the glenoid and the supraspinatus tendon. The release consisted of resection of all contractures within the rotator interval triangle, including the region of the coracohumeral ligament, the anterior capsule, the glenohumeral ligaments, and the subscapularis bursa. Further posterior or inferior release was individualized depending on the preoperative and intraoperative range of motion (ROM), avoiding unnecessary extensive release. When the inferior capsular release was performed, the working portal was switched to the posterior portal, and electrocautery was maintained within 10 mm of the glenoid rim to avoid axillary nerve damage [30]. The recovery of the ROM of the shoulder was verified at the end of the surgical procedure. Passive ROM exercises were initiated 12 h after surgery. Active and active-assisted ROM began 1 week postoperatively; strengthening exercises were delayed until 3 weeks postoperatively.

A multimodal analgesia strategy combining an oral nonsteroidal anti-inflammatory drug (i.e., diclofenac 75 mg every 12 h for 4 days) and an oral opioid (i.e., tapentadol 50 mg every 12 h for 6 weeks) was used in the absence of specific contraindications to improve postoperative pain and reduce the consumption of each agent [10].

### Functional and psychological assessment

Preoperatively, each patient was evaluated with the ROM assessment and the Constant-Murley score (CMS). At follow-up, each patient was evaluated with the 4-point subjective satisfaction scale (SSS) [9] (4 = excellent, 3 = good, 2 = fair, 1 = poor), the ROM assessment, the SF-12 questionnaire to measure the health status, the numerical rating scale (NRS) for the subjective assessment of pain, the CMS and the Hospital Anxiety and Depression Scale (HADS) [4]. Preoperative and postoperative patient’s assessment was performed by trained physicians, who were unaware of the diagnosis and treatment. The CMS was normalized for sex and age using the following formula: normalized CMS = (raw CMS/normal CMS) × 100 [19]. The HADS, which is used to assess the state of anxiety and depression in patients who have undergone a surgical operation, consists of two subscales, HADS-A and HADS-D for anxiety and depression, respectively; for each scale, final scores of ≤ 7, 8–10, and ≥ 11 were indicative of no significant anxiety or depression, borderline anxiety or depression, and clinical anxiety or depression, respectively [2, 13]. All patients were evaluated for intra- or postoperative complications and for their return to work and sports activities.

### Statistical analysis

All data were collected, measured, and reported within 1-decimal place accuracy. Uni- and multivariate linear regressions were performed on the whole population to test possible outcome predictors. The explanatory and confounding pre- and postoperative variables included in the analysis were sex (categorical), age (continuous), etiology (categorical), surgery side (categorical), dominant limb (categorical), comorbidities (categorical), time from surgery to return to work and sports (continuous), ROM (continuous), SF-12 (continuous), NRS (continuous), HADS (continuous), and follow-up (continuous). The SSS (continuous) and CMS (continuous) were treated as outcomes of the variables [8]. Only explanatory and confounding variables that showed a trend toward an association (e.g., *P* < 0.10) with the outcome of interest in the univariate analysis were inserted in the multiple regression analysis.

Post hoc power was calculated by considering the sample size, the observed effect size, and an *α*-value of 0.05; a post hoc power superior to 80% was considered appropriate. IBM SPSS Statistics software (version 26, IBM Corp., Armonk, NY, USA) and G*Power (version 3.1.9.2, Institut für Experimentelle Psychologie, Heinrich Heine Universität, Düsseldorf, Germany) were used for database construction and statistical analysis. A p value of less than 0.05 was considered significant.

## Results

Of the initial 85 patients, 7 were lost to follow-up, leaving 78 patients available for the final evaluation. The characteristics of the study population and the indications for the capsular release procedure are summarized in Table 1. There were 32 (41%) males and 46 (59%) females, with a mean age of 58.3 ± 9.3 years (range 30–80) at surgery. Of these patients, 6 (7.7%) performed sports activity. Among the postoperative frozen shoulder cases, the most common surgery was arthroscopic rotator cuff repair (10 cases, 32.2%); proximal humerus fracture (7 cases, 63.6%) was the most common cause of posttraumatic cases. The mean time of nonoperative management before surgery, which included oral steroids and physical therapy, was 4.7 ± 0.7 months (range 4–6). The mean time of postoperative physical therapy was 1.8 ± 0.5 months (range 1–3).

**Table 1:** Baseline characteristics of included patients

Patients (*n* = 78)	Mean ± SD (range) or *n* (%)
Gender	
Male	32 (41%)
Female	46 (59%)
Age at follow-up (years)	58.3 ± 9.3 (30–80)
Side	
Right	37
Left	41
Limb dominance	41 (52.6%)
Diabetes mellitus	12 (15.4%)
Hypothyroidism	5 (6.4%)
Physical therapy preop (months)	4.7 ± 0.7 (4–6)
Etiology	
Idiopathic	36 (46.2%)
Post-operative	31 (39.7%)
Post-traumatic	11 (14.1%)
Follow-up (months)	54.2 ± 22.3 (18–96)

*SD* Standard Deviation, *n* Number of cases

Functional and psychological outcomes after a mean follow-up of 54.2 ± 22.3 months (range 18–96) are shown in Table 2.

**Table 2:** Functional and psychological outcomes of the study population

SSS	3.7 ± 0.5 (1–4)
Return to work (months)	2.0 ± 0.9 (1–6)
Return to sport (months)	2.5 ± 0.5 (2–3)
CMS	88.9 ± 6.0 (57–98)
Mean increase in CMS	56.1 ± 8.3 (24–75)
SF-12	53.1 ± 1.4 (50–56)
HADS-A	2.5 ± 1.6 (0–9)
HADS-D	2.2 ± 1.3 (0–5)
NRS	2.2 ± 1.0 (0–6)
Worst pain over the last 24 h (NRS)	1.7 ± 1.3 (0–6)

Values are reported as mean ± SD (range)

*SSS* means subjective satisfaction scale, *CMS* Constant and Murley score, *HADS-A* Hospital anxiety and depression scale-anxiety, *HADS-D* Hospital anxiety and depression scale-depression, *NRS* numerical rating scale

Table 3 shows the differences between external rotation, flexion, and abduction ROM and CMS values before surgery and at follow-up. All outcomes showed a statistically significant improvement (all *p* < 0.001). Before surgery, the mean CMS was 36.9% that of sex- and age-matched healthy individuals, and all patients showed a CMS lower than the normative data. At the final follow-up visit, the mean CMS was 99.9% that of sex- and age-matched healthy individuals, and 49 (62.8%) patients showed a CMS equal to or higher than the normative data. The mean increase in the CMS was 56.1 ± 8.3 points (range 24–75). All patients returned to their previous level of work and sports activity. The mean SSS was 3.7 ± 0.5 (range 1–4), and 77 out of 78 (98.7%) patients stated that they were satisfied and would have the surgery again. Table 4 shows postoperative outcomes according to the etiology group.

**Table 3:** Differences in range of motion and Constant and Murley score between pre- and postoperative values

Outcomes	Preoperative mean ± SD	Postoperative mean ± SD	p-value	95% CI	SED
External rotation 1	41.8 ± 4.5	63.9 ± 9.2	** < *0.001***	***− 24.39 to − 19.71***	***1.17***
External rotation 2	41.8 ± 4.5	74.2 ± 8.9	** < *0.001***	***34.67 to − 30.21***	***1.13***
Flexion	109.2 ± 25.1	168.1 ± 5.4	** < *0.001***	***− 64.61 to − 53.08***	***2.9***
Abduction	88.1 ± 27.5	167.4 ± 8.1	** < *0.001***	***− 85.83 to − 72.89***	***3.25***
CMS	32.8 ± 7.7	88.9 ± 6	** < *0.001***	***− 57.95 to − 54.20***	***0.94***

*CMS* Constant Murley Score, *SD* standard deviation

**Table 4:** Functional and psychological outcomes according to the etiology groups

	Idiopathic	Post-operative	Post-traumatic
SSS	3.7 ± 0.5	3.8 ± 0.4	3.5 ± 0.7
Return to work (months)	1.9 ± 0.8	2.2 ± 1	1.8 ± 0.8
Return to sport (months)	2.3 ± 0.6	2.5 ± 0.5	2.7 ± 0.6
External rotation 1	65.8 ± 6.5	61.9 ± 11.4	62.7 ± 9.0
External rotation 2	76.1 ± 5.8	72.4 ± 11.4	73.2 ± 9.0
Flexion	166.7 ± 4.8	169.4 ± 5.7	169.1 ± 5.4
Abduction	169.7 ± 7.3	164.5 ± 7.7	168.2 ± 9.8
CMS	90.0 ± 4.1	88.2 ± 7.6	88.7 ± 6.6
Mean increase in CMS	58.9 ± 7.6	53.9 ± 8.9	53.1 ± 6.3
SF-12	52.9 ± 1.3	53.6 ± 1.4	52.3 ± 1.7
HADS-A	2.3 ± 1.4	2.5 ± 1.9	2.9 ± 0.9
HADS-D	2.3 ± 1.4	2.2 ± 1.4	2.0 ± 0.9
NRS	2.0 ± 1.2	2.3 ± 1.0	2.5 ± 0.9
Worst pain over the last 24 h (NRS)	1.7 ± 1.4	1.6 ± 1.2	2.1 ± 1.6

Values are reported as mean ± SD (range)

*SSS* means subjective satisfaction scale, *CMS* Constant and Murley score, *HADS-A* Hospital anxiety and depression scale-anxiety, *HADS-D* Hospital anxiety and depression scale-depression, *NRS* numerical rating scale

As shown in Table 5, the postoperative CMS values were directly related to the preoperative CMS values, the idiopathic etiology, and as expected, the postoperative SSS and SF-12 values. The multivariate analysis confirmed the association between a higher postoperative CMS and the idiopathic etiology of a frozen shoulder (*p* = 0.004, *β* = 3.971). The postoperative SF-12 values were affected by a lower postoperative NRS (*p* = 0.023, *β* = − 0.066).

**Table 5:** Linear Regression analysis: factors affecting postoperative Constant and Murley score

	*p*	*β*
SSS	0.026	0.06
Preoperative CMS	0.013	0.078
Postoperative SF-12	0.011	0.082
Idiopathic etiology	0.004	0.010

*SSS* means subjective satisfaction scale, *CMS* constant and Murley score, *NRS* numerical rating scale

No intraoperative complications occurred. Postoperatively, four patients (5.1%) were treated with intra-articular steroid injections to manage residual symptoms. One patient (1.3%) showed a persistent frozen shoulder and underwent a new successful arthroscopic capsular release. The patient had a CMS of 57 points and 50° external rotation in adduction, 60° external rotation in abduction, 170° flexion, 160° abduction, and internal rotation at the lumbo-sacral level at the last follow-up; he was a 52-year-old man with a frozen shoulder after an arthroscopic reduction and fixation of a greater tuberosity fracture.

## Discussion

The most important finding of the present study was that the overall functional and psychological outcomes of the arthroscopic capsular release after a mean 4.5-year follow-up were successful. High patient satisfaction and statistically significant ROM and CMS recovery were reported. All patients returned to their previous level of work and sports activity. Higher postoperative CMS values were associated with idiopathic frozen shoulder cases.

To our knowledge, the study population in the present article represents one of the largest and longest series of frozen shoulder cases treated with arthroscopic capsular release, and overall, the successful results we reported confirm what Sivasubramanian et al. [30] found in their recent systematic review regarding the technique to be adopted. Indeed, the authors compared the clinical outcomes of a less extensive and patient-tailored release versus more extensive releases such as a 360-degree or posterior capsular release and reported that less extensive releases resulted in better functional scores. Furthermore, the less extensive release overcomes concerns of the more extensive techniques, such as an increased surgical time, technical difficulties, and complications [22].

In the current study, patients showed a mean CMS recovery of 56.1 points with a mean postoperative CMS of 88.9; their postoperative CMS values were 99.9% of those of sex- and age-matched healthy individuals. Jerosh et al. [17] analyzed 28 patients and found a mean postoperative CMS of 85 points with a mean CMS recovery of 41 points at a mean follow-up of 22 months. Ranalletta et al. [27] reported a significant improvement in pain and CMS in 32 patients who described their result as good to excellent after a mean follow-up of 63 months; the authors also reported a mean postoperative flexion, abduction, and external rotation of 171°, 164°, and 66°, respectively, which concur with those reported in the current work.

In the current study, successful postoperative psychological outcomes were reported with a mean HADS-A and HADS-D scores of 2.5 and 2.2, respectively, and these scores were not correlated with the postoperative functional scores. It was reported [5] that in frozen shoulder patients, the prevalence of anxiety and depression was 24.2% and 28.2%, respectively, and the symptoms could be associated with more severe preoperative pain and functional restriction. Overall, these results suggest the importance of assessing psychological symptoms among frozen shoulder patients and that normal HADS values can be achieved after capsular release.

In the current study, interestingly, better postoperative CMS values were associated with the idiopathic etiology. The pathophysiology of frozen shoulder is correlated with an inflammatory-fibrotic cascade that leads to increased fibroblast proliferation with collagen fiber deposition, increased vascularization and new nerve growth. Collagen fibers adhere to the glenohumeral ligaments, tendons, and joint surfaces, causing contracture and joint stiffening. In this context, frozen shoulder is defined as idiopathic when history and examination cannot explain the development of the condition, and this is distinct from postoperative and posttraumatic etiologies. The cause of frozen shoulder remains largely unknown, and several risk factors have been identified, including diabetes, thyroid disorders, rheumatoid arthritis, gout, and Parkinson’s disease [29]. A meta-analysis showed that the prevalence of diabetes mellitus in a population with a frozen shoulder can reach 30% and that diabetic patients were 5 times more likely to develop a frozen shoulder [11]. In the present study, a prevalence of diabetes mellitus and hypothyroidism of 15.4% and 6.4%, respectively, was reported. Interestingly, the prevalence of diabetes mellitus in the general population is 5.9% [7], and our data seem to confirm its possible pathogenetic role, but no significant association with postoperative outcomes was found, as recently suggested by Mertens et al. [23] in a short term multicenter observational study. Systematic reviews [6, 24] confirmed conflicting evidence regarding diabetes mellitus as a possible prognostic factor for influencing clinical outcomes in patients with frozen shoulder.

In the current study, only one arthroscopic capsular release revision procedure was reported to manage persistent symptoms in a patient with a postoperative frozen shoulder. Interestingly, Boutefnouchet et al. [1] reported that the rate of revision capsular release was four times higher among patients with postoperative frozen shoulder when compared to idiopathic cases. Moreover, arthroscopic capsular release in postoperative and posttraumatic patients has been associated with worse outcomes than idiopathic cases. Holloway et al. [14] compared outcomes after arthroscopic release in idiopathic, posttraumatic and postoperative groups; the authors reported that patients with postoperative etiology had significantly worse scores for pain, satisfaction and functional activity.

The major limitations of the current study are its retrospective nature and the lack of a control group; a group undergoing conservative treatment would have provided an interesting comparison with outcomes after arthroscopic release. Similarly, the presence of a control group undergoing no treatment at all would have provided an interesting comparison between the rate of spontaneous recovery of stiffness with that of stiffness recovery after arthroscopic release. However, the lack of a control group may be mitigated by the predominance in the current study population of posttraumatic and postoperative cases that more often show poor prognosis requiring surgical treatment and a lower probability of spontaneous recovery. The lack of preoperative administration of psychological evaluation scales that were used at follow-up hinders the ability to draw firm conclusions from the reported results. As an additional weakness, the current study did not include a clinical psychiatric evaluation other than the patient’s self-assessment. Therefore, self-report response bias cannot be excluded; moreover, nor can the possibility that psychological well-being and functional recovery may be influenced by preexisting mental health conditions. The prospective nature of the data collection methods, the use of validated and standardized functional and psychological assessments, the statistical reliability produced by the regression analyses, and the sample size and follow-up being comparable to the largest and longest series available [30] represent the considerable strengths of the present study. The findings of the current study may help surgeons identify patients who will benefit most from a patient-tailored arthroscopic capsular release and should be discussed preoperatively especially with the patient at risk for a less successful outcome.

## Conclusions

In summary, high patient satisfaction and statistically significant ROM and CMS recovery can be achieved after arthroscopic capsular release to manage frozen shoulder. Better functional outcomes are expected when the etiology is idiopathic.

## Data Availability

Data supporting the results of this study are available within the article. Raw data are available from the corresponding author upon reasonable request.
